# Enhancing the Performance of MoS_2_ Field-Effect Transistors Using Self-Assembled Monolayers: A Promising Strategy to Alleviate Dielectric Layer Scattering and Improve Device Performance

**DOI:** 10.3390/molecules29173988

**Published:** 2024-08-23

**Authors:** Li Cao, Junqing Wei, Xianggao Li, Shirong Wang, Guoxuan Qin

**Affiliations:** 1 School of Chemical Engineering and Technology, Tianjin University, Tianjin 300072, China; 2 Collaborative Innovation Center of Chemical Science and Engineering, Tianjin 300072, China; 3 Tianjin Key Laboratory of Imaging and Sensing Microelectronic Technology, School of Microelectronics, Tianjin University, Tianjin 300072, China

**Keywords:** dipole moment, field-effect transistors, MoS_2_, mobility, phonon scattering, self-assembled monolayers

## Abstract

Field-effect transistors (FETs) based on two-dimensional molybdenum disulfide (2D-MoS_2_) have great potential in electronic and optoelectronic applications, but the performances of these devices still face challenges such as scattering at the contact interface, which results in reduced mobility. In this work, we fabricated high-performance MoS_2_-FETs by inserting self-assembling monolayers (SAMs) between MoS_2_ and a SiO_2_ dielectric layer. The interface properties of MoS_2_/SiO_2_ were studied after the inductions of three different SAM structures including (perfluorophenyl)methyl phosphonic acid (PFPA), (4-aminobutyl) phosphonic acid (ABPA), and octadecylphosphonic acid (ODPA). The SiO_2_/ABPA/MoS_2_-FET exhibited significantly improved performances with the highest mobility of 528.7 cm^2^ V^−1^ s^−1^, which is 7.5 times that of SiO_2_/MoS_2_-FET, and an on/off ratio of ~10^6^. Additionally, we investigated the effects of SAM molecular dipole vectors on device performances using density functional theory (DFT). Moreover, the first-principle calculations showed that ABPA SAMs reduced the frequencies of acoustic and optical phonons in the SiO_2_ dielectric layer, thereby suppressing the phonon scattering to the MoS_2_ channel and further improving the device’s performance. This work provided a strategy for high-performance MoS_2_-FET fabrication by improving interface properties.

## 1. Introduction

Two-dimensional (2D) semiconductors, including transition metal dichalcogenides (TMDCs), are highly promising for creating transparent, flexible, and wearable electronics and optoelectronics, owing to their ultra-low dimensions, high carrier mobility, and highly effective light absorption capability [1,2,3]. Among these TMDCs, molybdenum disulfide (MoS_2_) is one of the most promising semiconductor materials, possessing typical n-type characteristics and high stability [4,5]. In theory, MoS_2_-based field-effect transistors (FETs) are expected to achieve a significant on/off ratio (>10^9^) and a room-temperature mobility of 410 cm^2^ V^−1^ s^−1^ [6]. Different from the Si semiconductor, the 2D-MoS_2_ has a smooth surface without any dangling bonds; thus, the channel material MoS_2_ and the dielectric layer are bonded via van der Waals’ force. However, strong phonon scattering at the semiconductor dielectric layer interface has hindered experimental results from achieving these theoretical expectations [7].

Until now, MoS_2_ has been combined with high-κ dielectric materials including aluminum oxide (Al_2_O_3_) and hafnium oxide (HfO_2_) for the fabrication of field-effect transistors (FETs) [8]. Unfortunately, the amorphous character of most dielectrics and adverse dielectric/MoS_2_ interactions pose a challenge for eliminating charge-carrier scattering sites and traps [9,10]. An alternative and attractive approach is to introduce ripples into the lattice structure of the MoS_2_, where the lattice distortions can reduce electron–phonon scattering in 2D materials and thereby enhance the charge carrier mobility [11]. However, few reports have investigated high-performance MoS_2_ FETs based on the traditional and cost-effective SiO_2_ dielectric layer due to its low-κ nature and the associated charge traps [12,13,14]. This limitation has hindered the further development of MoS_2_-based FETs for next-generation nano-electronic devices.

The self-assembled monolayers (SAMs) of organic molecules have garnered considerable attention in surface and interface engineering owing to their ability to spontaneously form ultrathin molecular films at the substrate interface via chemical or physical processes [15]. By altering the functional groups, SAMs allow the facile tuning of surface energy, dipole moment, and chemical reactivity, making them a versatile tool for tailoring surface properties [16,17,18,19]. Remarkably, SAMs with amine-based functionalities have been shown to promote electron accumulation or enhance the charge transfer between functional SAMs and other materials, thereby enabling the more precise tuning of their characteristics [20,21,22,23,24].

In this study, we presented SiO_2_/SAMs/MoS_2_ hybrid-structure FETs and investigated three different types of SAMs to modulate the contact interface property of SiO_2_/MoS_2_, including ABPA, PFPA, and ODPA. The performances of three types of SiO_2_/SAMs/MoS_2_-FETs were studied and the SiO_2_/ABPA/MoS_2_-FET showed the best performances with a high carrier mobility of 528.7 cm^2^ V^−1^ s^−1^ and an on/off ratio of ~10^6^, which was better than that of SiO_2_/MoS_2_-FETs without SAMs (mobility of ~70.32 cm^2^ V^−1^ s^−1^, on/off ratio of ~10^3^). After inducing ABPA, the surface potential of SiO_2_ was calculated via density functional theory (DFT). According to the simulations, the interfacial potential of the SiO_2_/MoS_2_ interface was positively enhanced by ABPA and, thus, contributed to more charge accumulation and improved carrier mobility. Additionally, the SAMs could effectively reduce phonon scattering from the SiO_2_ dielectric layer and SiO_2_/MoS_2_ interface, which provided a promising strategy for the fabrication of high-performance MoS_2_-FETs.

## 2. Results and Discussion

**Figure 1:**
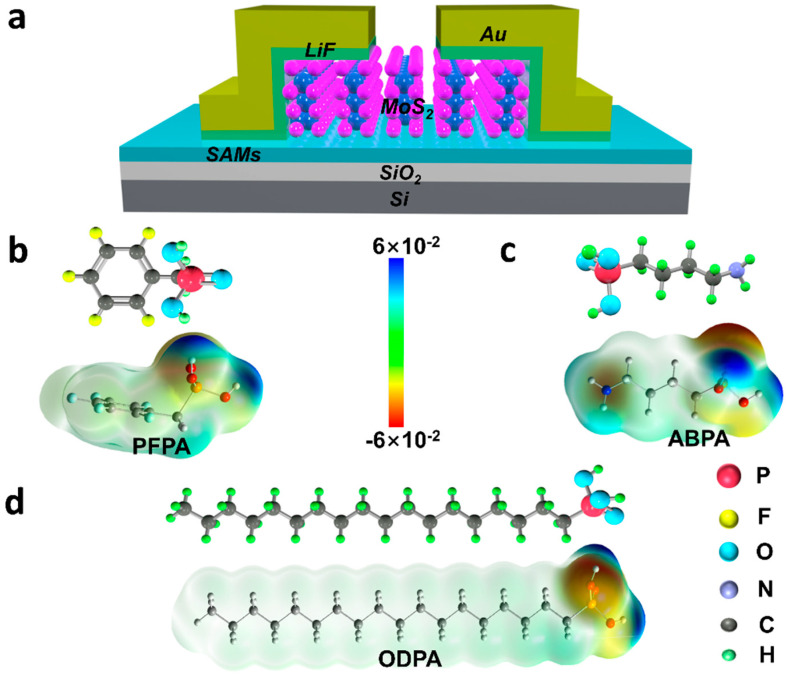
The structure of SiO_2_/SAM/MoS_2_-FET. (**a**) Schematic illustration of MoS_2_-FET. The chemical structures and electrostatic surface potential (ESP) maps of three types of SAMs: (**b**) PFPA, (**c**) ABPA, and (**d**) ODPA.

The MoS_2_-FET device structure is depicted in Figure 1a, featuring a common bottom-gate configuration. Mechanically exfoliated multilayer MoS_2_ films served as the semiconductor channel, and the LiF (3 nm)/Au (60 nm) acting as the source/drain contacts were deposited via vacuum evaporation. Recent studies have shown that the contact resistance can be significantly reduced with the incorporation of the LiF layer [25]. The substrate used in this study was heavily p-doped Si (~0.05 Ω·cm), which also served as the common bottom-gate electrode. And, a ~300 nm SiO_2_ layer was utilized as the dielectric layer.

**Figure 2:**
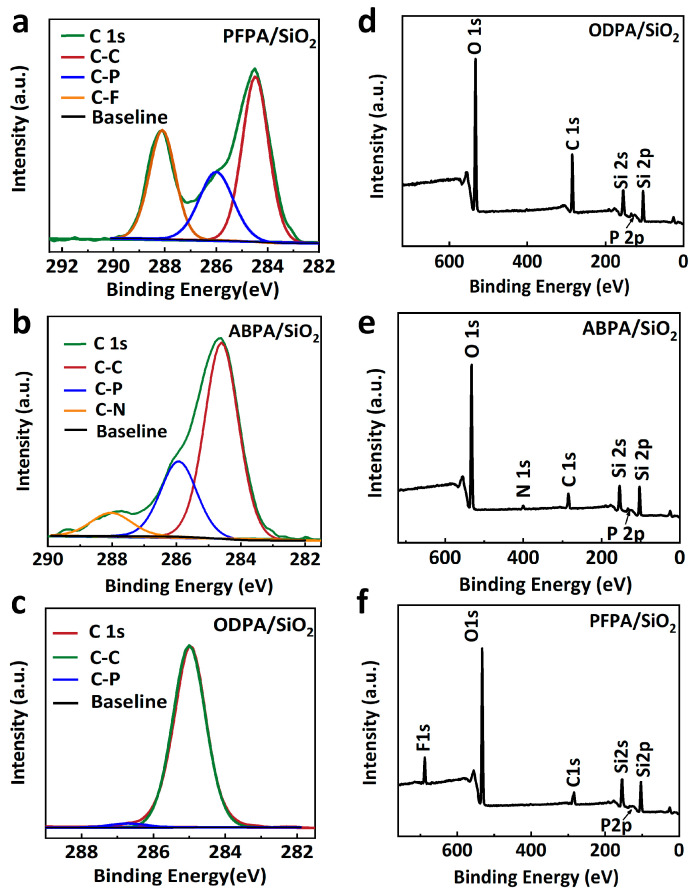
X-ray photoelectron spectroscopy (XPS) analysis of C 1s spectra in (**a**) PFPA/SiO_2_, (**b**) ABPA/SiO_2_, and (**c**) ODPA/SiO_2_, respectively. XPS survey spectra of (**d**) PFPA/SiO_2_, (**e**) ABPA/SiO_2_, and (**f**) ODPA/SiO_2_, respectively.

**Figure 3:**
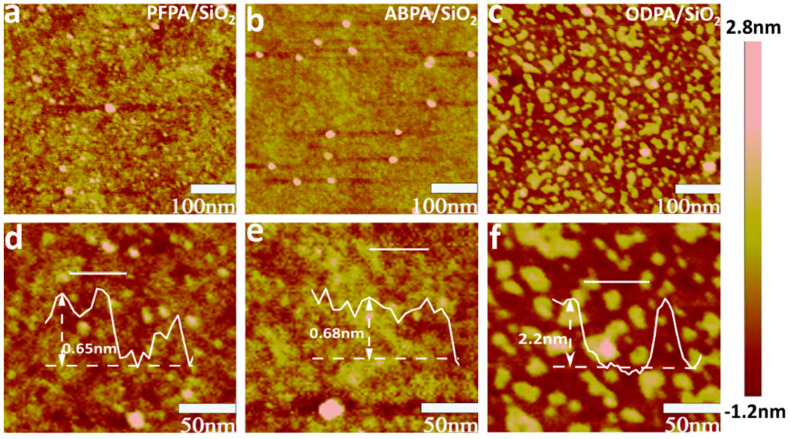
Surface morphologies of (**a**) SiO_2_/PFPA, (**b**) SiO_2_/ABPA, and (**c**) SiO_2_/ODPA. Section analyses of SAM: (**d**) PFPA, (**e**) ABPA, and (**f**) ODPA.

In the present study, three types of phosphonic acid coupling agents were selected based on their dipole moments and polarity, including (perfluorophenyl) methyl phosphonic acid (PFPA), (4-aminobutyl) phosphonic acid (ABPA), and octadecylphosphonic acid (ODPA). The electrostatic surface potential (ESP) maps of the self-assembled monolayers (SAMs) revealed the variable dipole moments associated with their functional groups. Due to the significant electronegativity of fluorine atoms, PFPA (Figure 1b) can enhance the hole accumulation at the dielectric layer interface, which is beneficial for p-type transport. On the other hand, ABPA containing the NH_2_ functional group (Figure 1c) and ODPA terminated with CH_3_ (Figure 1d) exhibit certain electron-donating properties, which can enhance the electron accumulation at the dielectric layer interface to a certain extent and facilitate n-type transport. During the modification processes (Figure S1), PAs reacted with hydroxyl groups and the bridging oxygen on the surface of SiO_2_ [26]. The chemical states of the carbon atoms in the SAMs molecules were examined using X-ray photoelectron spectroscopy (XPS) analysis, as shown in Figure 2a–c. The binding energy of each sample was calibrated with respect to the peaks at 103.6 eV corresponding to the Si 2p of the pristine SiO_2_/Si substrate. The C1s peaks of the C-P bonds in PFPA, ABPA, and ODPA were located at 286.1 eV, 286.0 eV, and 286.4 eV, respectively. The slight shifts observed in the C1s peaks may be attributed to the differences in the polarity of the SAM molecules [21]. Moreover, the C1s peak of the C-N group was located at 288 eV for ABPA, and by calculating the fitted areas of the C-N and C-P peaks in Figure 3b, we found that the carbon content ratio of C-P to C-N was approximately 1.5, which is higher than the original ratio of C-P/C-N = 1. This deviation may have been caused by the decomposition of C-N bonds due to irradiation by X-rays during the XPS measurements. In order to gain further insights into the chemical states of the elements within the SAMs, X-ray photoelectron spectroscopy (XPS) analysis was conducted. As illustrated in Figure 2d–f, the survey spectra of the three types of SAMs were characterized by peaks that correspond to their respective elements. Evidently, as depicted in the figure, the long alkyl chain of ODPA leads to a significantly stronger diffraction intensity of its C1s compared to that of ABPA and PFPA. Additionally, the N1s peak was observed at 400.4 eV for ABPA-SiO_2_, whereas the F1s peak was located at 688.3 eV for PFPA-SiO_2_. All of the aforementioned findings further validate that the three phosphonic acid molecules have conducted effective self-assembly on the surface of SiO_2_.

Atomic force microscopy (AFM) measurements were conducted to investigate the morphology of SAM films. The SiO_2_/Si substrates had high flatness and consistent SAM films after being modified with PFPA (Figure 3a), ABPA (Figure 3b), and ODPA (Figure 3c). According to the analysis of AFM morphologies, the root-mean-square (RMS) roughness of PFPA-SiO_2_, ABPA-SiO_2_, and ODPA-SiO_2_ are 0.43, 0.33, and 0.84 nm, respectively. Figure 3d–f depicted the thicknesses of the self-assembled monolayers (SAMs) of PFPA, ABPA, and ODPA, which measured approximately 0.65 nm, 0.68 nm, and 2.2 nm, respectively. These measurements were in agreement with the height of a single molecule, thus indicating the formation of stable and uniform PA monolayers on the surface of SiO_2_ through self-assembly [27]. The pink bulges represent multilayer phosphonic acid (PA) molecule aggregations because of the cross-link interactions with other SAM precursors in a liquid environment [28]. Additionally, we conducted water contact angle tests on the three self-assembled monolayers, as shown in Figure S2. The contact angles were 108°(ODPA/SiO_2_), 103°(PFPA/SiO_2_), and 69°(ABPA/SiO_2_), respectively. ODPA/SiO_2_ and PFPA/SiO_2_ exhibit excellent hydrophobicity, mainly attributed to the long alkyl chain of ODPA and the F elements in PFPA. Correspondingly, due to the presence of the -NH_2_ head group in ABPA, it shows a certain degree of hydrophilicity. Overall, all three self-assembled molecules form stable and uniform monolayers on the SiO_2_ surface.

**Figure 4:**
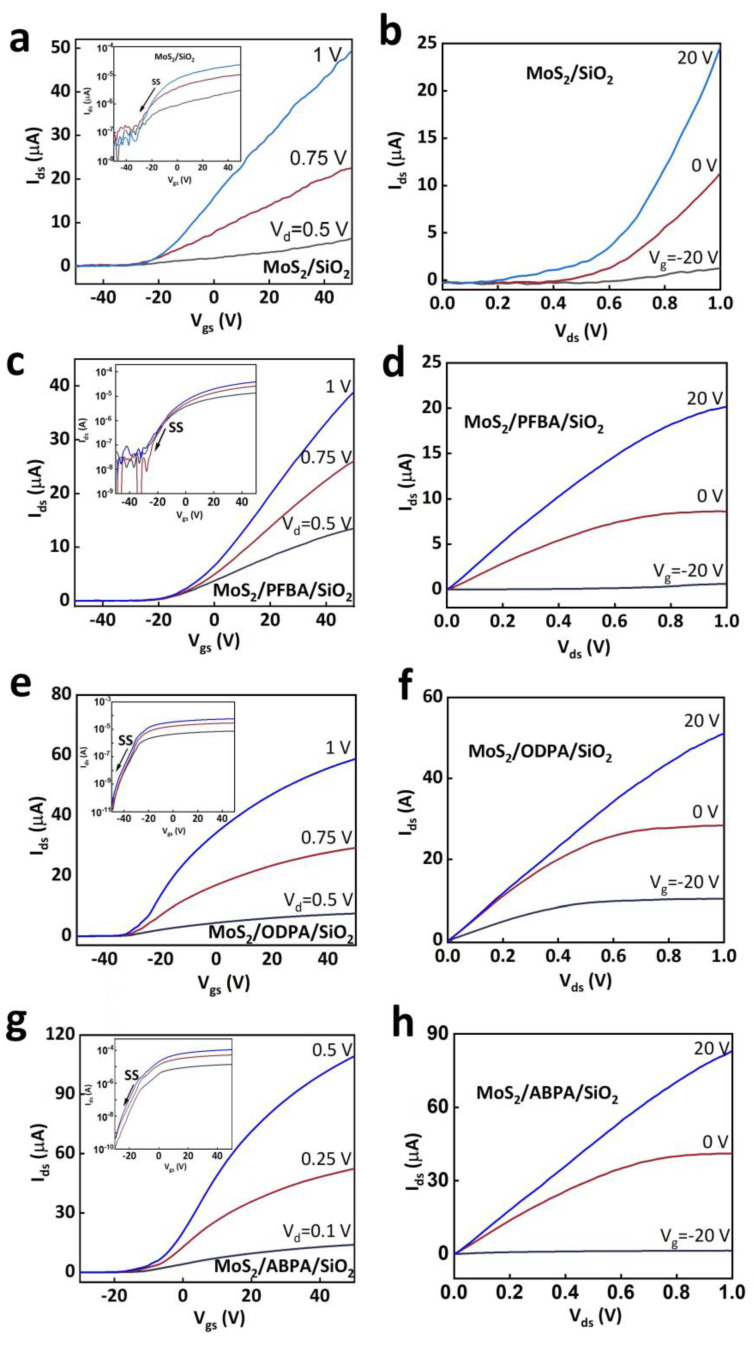
The transfer and output properties of the MoS_2_-FETs. (**a**,**b**) SiO_2_/MoS_2_-FET (without SAMs) (**c**,**d**) SiO_2_/PFPA/MoS_2_-FET, (**e**,**f**) SiO_2_/ODPA/MoS_2_-FET, and (**g**,**h**) SiO_2_/ABPA/MoS_2_-FET.

The performances of MoS_2_-FET devices modified with three different types of SAMs and a SiO_2_/MoS_2_-FET (without SAM) are presented in Figure 4. The drain current (I_ds_) exhibited a monotonic increase with the gate voltages (V_g_) ranging from −40 to +40 V, indicating a typical n-channel transistor behavior. The on/off ratio and subthreshold swing (SS) of the SiO_2_/MoS_2_-FET device were extracted to be ∼1 × 10^3^ and ~12 V/dec from the saturation transfer characteristics as shown in the inset of Figure 4a (with a semi-log scale). The PFPA-modified device (Figure 4c) exhibited an improved on/off ratio of 2 × 10^3^ and an SS of ~10 V/dec. Similarly, the ODPA-modified device (Figure 4e) displayed an on/off ratio of ~1.8 × 10^6^ and an SS of approximately 5 V/dec, and the ABPA-modified device showed an on/off ratio of ~1.7 × 10^6^ (Figure 4g), and the lowest SS of ~4 V/dec among these. Furthermore, we investigated the influence of self-assembled molecules on the interface defect density (D_IT_); the evaluation of D_IT_ can be experimentally carried out or theoretically estimated according to Equation (1) [29]:

(1) $$SS=\frac{kTln10}{e}(1+\frac{e^{2}}{C_{S}}D_{IT})$$

where *k* is the Boltzmann constant, *T* is the temperature, *e* is the elementary charge, *C_S_* is the specific capacitance of the gate dielectric, and *D_IT_* is the interfacial defect density. Through this relation, these associated defect densities were estimated to be ∼1.43 × 10^14^ eV^−1^ cm^−2^ (SiO_2_/MoS_2_-FET), 1.19 × 10^14^ eV^−1^ cm^−2^ (PFPA), 5.91 × 10^13^ eV^−1^ cm^−2^ (ODPA), and 4.71 × 10^13^ eV^−1^ cm^−2^ (ABPA), respectively. These results indicate that all three self-assembled molecules can, to a certain extent, reduce the interface defect density, decrease the capture of carriers, and enable more efficient current switching between the on-state and off-state, thereby achieving a higher on/off current ratio compared to the unmodified device.

The electron field-effect mobility of all four types of MoS_2_-FETs can be calculated from the transfer characteristics and Equation (2) [11],

(2) $$\mu =\frac{1}{C_{ox}}\frac{L}{W}\frac{1}{V_{ds}}g_{m}$$

where *C_ox_* is the capacitance (accumulation capacitance) per unit area, *L* and *W* are the channel length and width respectively, and *g_m_* is the transconductance of the FET. Firstly, the transconductances of the four types of MoS_2_-FETs were calculated for the SiO_2_/MoS_2_-FET (Figure S3a) and those modified with PFPA (Figure S3b), ODPA (Figure S3c), and ABPA (Figure S3d), respectively. As shown in Figure S4, the calculated highest and average mobilities were 70.32 cm^2^ V^−1^ s^−1^ and 54.9 ± 12.3 cm^2^ V^−1^ s^−1^ (SiO_2_/MoS_2_-FET), 64.3 cm^2^ V^−1^ s^−1^ and 57.5 ± 6.8 cm^2^ V^−1^ s^−1^ (PFPA), 172.6 cm^2^ V^−1^ s^−1^ and 161.9 ± 9.5 cm^2^ V^−1^ s^−1^ (ODPA), and 528.7 cm^2^ V^−1^ s^−1^ and 511.1 ± 18.2 cm^2^ V^−1^ s^−1^ (ABPA), respectively. 

**Figure 5:**
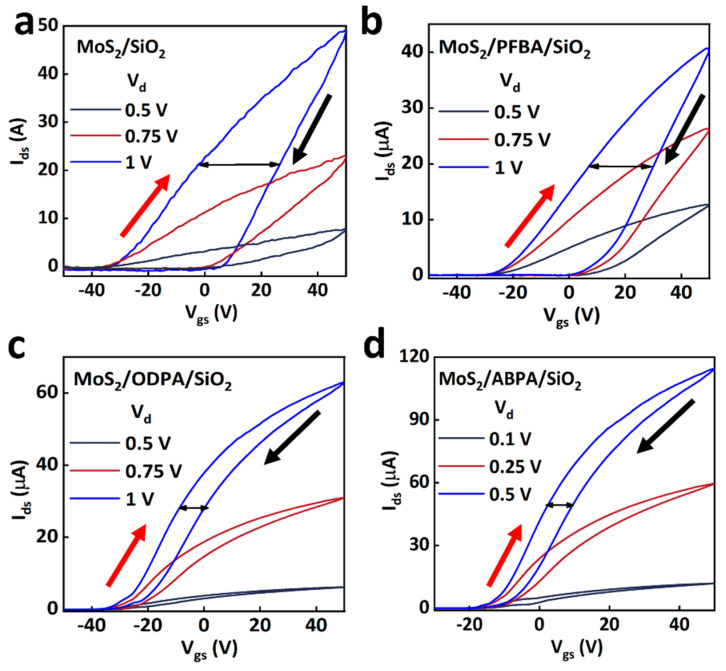
The hysteresis properties of the MoS_2_-FETs: (**a**) SiO_2_/MoS_2_-FET, (**b**) PFPA, (**c**) ODPA, and (**d**) ABPA.

Additionally, the hysteresis properties of these devices were analyzed according to the threshold voltage shift (Figure 5, The red and black arrows in the figure represent the forward and reverse scans respectively), and they were strongly influenced by the dielectric layer and atmospheric conditions [21]. Specifically, the SiO_2_/MoS_2_-FET (Figure 5a) and PFPA-(Figure 5b), ODPA-(Figure 5c), and ABPA-(Figure 5d) modified devices exhibited threshold voltage shifts of 26.5 V, 30.9 V, 8 V, and 10 V, respectively. The SiO_2_/ODPA/MoS_2_ and SiO_2_/ABPA/MoS_2_ FETs had smaller hysteresis compared to the MoS_2_/SiO_2_ and SiO_2_/PFPA/MoS_2_ FETs. And, the obvious hysteresis in the SAMS-modified FETs might be attributed to the adsorption of H_2_O or O_2_ molecules at the channel surface and the dangling bonds with SAMs [30].

**Figure 6:**
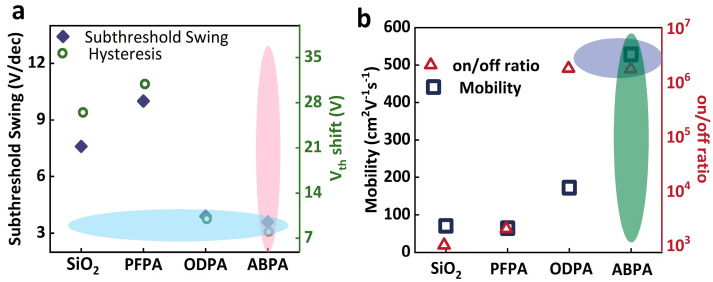
The performance comparisons of the SiO_2_/SAMs/MoS_2_-FETs; (**a**) the subthreshold swing and hysteresis properties. (**b**) The mobility and on/off ratio of all devices.

The comparisons of the semiconductor performances among the four types of MoS_2_-FETs are presented in Figure 6. In contrast to SiO_2_/MoS_2_-FET, the SS and hysteresis of SiO_2_/PFPA/MoS_2_-FET increased, while those of SiO_2_/ODPA/MoS_2_-FET and SiO_2_/ABPA/MoS_2_-FET decreased significantly (As shown in the blue and pink shaded parts in Figure 6a). Remarkably, the mobility and on/off ratio were enhanced, and the ABPA-treated sample exhibited the highest mobility and on/off ratio among all the samples investigated (As shown in the purple and green shaded parts in Figure 6b), surpassing the mobility values reported in previous studies of MoS_2_-FETs based solely on SiO_2_ dielectric layers [31].

**Figure 7:**
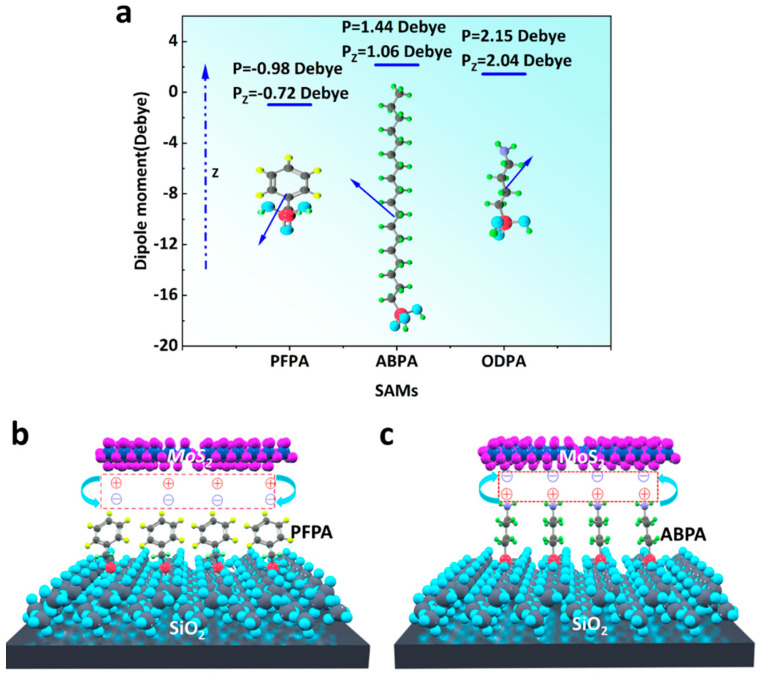
Calculation results: (**a**) Calculation of dipole moments of the SAMs molecules. Schematic of SAM-induced charged surface and formation of a space-charge layer at the interface for (**b**) PFPA and (**c**) ABPA.

Moreover, we analyzed the reasons for the device performance improvements and working mechanisms of SAMs in MoS_2_ FETs. In general, the enhancement of mobility has been explained by taking the electrostatic potential (*V_SAMs_*) generated by SAMs into consideration [32]. The evaluation of the potential can be experimentally carried out or theoretically estimated according to the Helmholtz equation:

(3) $$V_{SAMs}=\frac{N_{SAM}P_{z}}{\varepsilon_{0}\varepsilon_{SAM}}$$

where *N_SAMs_* is the molecular density of the SAMs (the number of molecules per unit area), *P_Z_* is the perpendicular component of the dipole moment of the SAMs, *ε_0_* is the vacuum permittivity, and *ε_SAM_* is the relative permittivity of the SAMs. In the estimation, we assumed that N_SAMs_ was approximately equal to 4.55–5.4 × 10^14^ cm^−2^, and *ε_SAM_* was 2~3 [33,34]. The *P_Z_* values of PFPA, ABPA, and ODPA were calculated and are shown in Table 1, according to the density functional theory (DFT) method at the B3LYP/D95 level using the model compounds (Figure 7a).

Evidently, the highest electrostatic potential (*V_SAMs_*) was induced by ABPA-SAMs among three molecules, and the corresponding *V_SAMs_* formed a space-charge layer at the semiconductor–dielectric interface. As we previously stated, the PFPA-modified substrate had a negative surface potential, and the existing electronic coupling between MoS_2_ and PFPA-SAMs resulted in a positive space-charge layer in the MoS_2_ semiconducting layer at the interface (Figure 7b). Electron transport in the MoS_2_ semiconducting layer could be reduced because the generated positive charge acted as trap sites for electrons injected from the drain electrode. Thus, a larger gate voltage was required to counteract the effects of the generated positive charge by PFPA-SAMs, which made the carrier mobility lower and also increased the hysteresis of the MoS_2_-FETs. On the contrary, the ABPA-SAMs exhibited a higher positive surface potential compared to the ODPA-SAMs. The magnitude of this difference was quantified as +800 mV for *V_SAMs_* = (*V_ABPA_* − *V_ODPA_*). The electronic coupling between MoS_2_ and ABPA-SAMs contributed to the creation of a more extensive negative space-charge layer in the MoS_2_ semiconducting layer at the interface (Figure 7c). This generated negative space-charge layer had a profound impact on carrier migration by enhancing it effectively. To promote the accumulation of charges in the dielectric layer during n-channel operation, it was crucial to create negative charges within the MoS_2_ semiconducting layer. This was achieved through the generation of a space-charge layer, which was brought about by the action of SAMs. Our analysis suggested that the development of this space-charge layer was a necessary condition for realizing the enhanced performance of MoS_2_ FETs.

**Figure 8:**
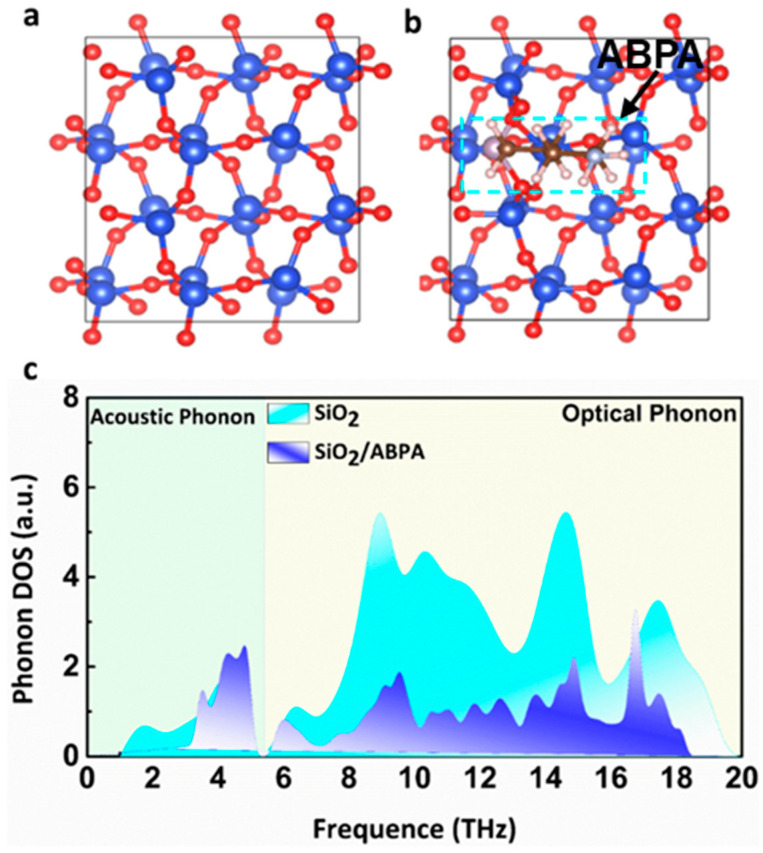
DFT-simulated relaxed structures of (**a**) unmodified SiO_2_ and (**b**) ABPA-modified SiO_2_. (**c**) Calculated phonon DOS in unmodified SiO_2_ and ABPA-modified SiO_2_, with frequencies of acoustic (optical) phonons.

To validate the effect of self-assembled molecules on the phonon scattering of the dielectric layers, we carried out density functional theory (DFT) calculations of SiO_2_ and ABPA-modified SiO_2_, and the computational methods of the phonon density of states (DOS) are shown in the Supplementary Materials (SII). The SiO_2_-terminated surface along the (101) direction was considered as a model atomic structure of unmodified SiO_2_ (Figure 8a), where an ABPA molecule was bound to the SiO_2_ (101) surface, which was modeled as modified SiO_2_ (Figure 8b). As shown in Figure 8c, phonons were renormalized due to the interaction between SiO_2_ and ABPA after binding. An obvious decrease in the frequency of acoustic phonons was observed and a renormalization of optical phonons was as well. This renormalization of optical phonons across a wider range of frequencies weakens their intensity due to the breaking of degeneracy induced by the formation of a covalent bond with ABPA-SiO_2_. The estimated effective density of scattering provided insight into the impact of renormalized phonons on mobility [35]. These renormalized phonons dramatically decrease the effective density of scattering in SiO_2_, thus resulting in strongly suppressed electron–phonon scattering and a high-mobility MoS_2_ FET based on the dielectric layer of SiO_2_.

## 3. Conclusions

To summarize, we presented a straightforward approach to modulate the insulating layer of SiO_2_ in MoS_2_ FETs using self-assembled monolayers (SAMs), resulting in an impressive transistor performance. Our results demonstrated that the dipole moments of SAMs gave rise to varying values and orientations of space-charge layers. Notably, the PFPA-modified substrate generated a positive space-charge layer, leading to larger hysteresis and lower mobility in MoS_2_ FETs compared to ODPA- and ABPA-modified substrates. Conversely, the ABPA-modified device exhibited high mobility up to 528.7 cm^2^ V^−1^ s^−1^, surpassing the mathematically predicted limit for the mobility of n-type MoS_2_ FETs. This exceptional mobility was primarily attributed to the formation of a larger and more negative space-charge layer, which promoted the accumulation of charges in the dielectric layer, as well as a reduction in phonon scattering of the insulating layer. These findings underline the potential of our approach for high-performance electronic devices, which is compatible with existing 2D semiconductor methods and offers a straightforward method for the manufacturing of 2D semiconductor-based devices.

## 4. Experimental Section

Device Fabrication: FETs in the bottom-gate configuration were fabricated using contact photolithography on the top of p-doped Si substrate with 300 nm of thermally grown SiO_2_ (C_ox_ = 11.7 nF/cm^2^). The SiO_2_/Si substrates modified with three types of phosphonic acid molecules (the modification methods are shown in detail in the Supplementary Materials (SI)). MoS_2_ layers were then fabricated on the Si/SiO_2_/SAMs layer via the mechanical exfoliation method. The source and drain regions were patterned on the MoS_2_ layers via the electron-beam lithography (EBL, Raith e-Line Plus) system. The channel dimensions were 10 mm in width and 10 mm in length. Finally, a 3 nm LiF and 60 nm Au metal layer were deposited via e-beam evaporation, followed by a lift-off process to form the source/drain electrodes.

Instruments and Measurements: The topography of SAM films was characterized by AFM (Bruker Dimension Icon, Billerica, MA, USA). The quality of the SiO_2_/SAMs was characterized by XPS (Thermo Scientific ESCALAB 250Xi, Waltham, MA, USA). The electrical analyses of the MoS_2_ were conducted using a semiconductor parameter analyzer (Keithley 4200 SCS, Cleveland, OH, USA) under ambient conditions.

Materials: (Perfluorophenyl)methyl phosphonic acid (PFPA), (4-aminobutyl) phosphonic acid (ABPA), and octadecylphosphonic acid (ODPA) were purchased from Sigma-Aldrich, Shanghai, China.

## Figures and Tables

**Table 1:** DFT-calculated molecular dipole (*P*_z_) and calculated (*V_SAMs_*) surface potentials of PFPA-, ABPA-, and ODPA-SAMs.

SAMs	*P* (debye)	*P*_Z_ (debye)	*V_SAMs_* (mV)
PFPA	−0.98	0.72	−496 to −589
ABPA	2.15	2.04	+1405 to +1668
ODPA	1.44	1.06	+730 to+868

## Data Availability

Data are contained within the article and Supplementary Materials.

## Supplementary Materials

The following supporting information can be downloaded at https://www.mdpi.com/article/10.3390/molecules29173988/s1: The details of the modification process of SiO_2_ using SAMs. The details of computational methods in ABPA-modified devices. Figure S1. Schematic diagram of the reaction mechanism of phosphonic acids with the hydroxyl groups and bridging oxygen on the SiO_2_ surface. Figure S2. Transconductance of SAM-modified FETs. Refs. [36,37,38,39,40,41,42] are cited in supplementary file.
