# Supplementary material for: Enhancing the Performance of MoS2 Field-Effect Transistors Using Self-Assembled Monolayers: A Promising Strategy to Alleviate Dielectric Layer Scattering and Improve Device Performance
Source: Molecules. 2024 Aug 23;29(17):3988. doi: 10.3390/molecules29173988 (PMC11396459; doi:10.3390/molecules29173988)
Supplement: Supplementary file 1 [file molecules-29-03988-s001.zip › molecules-3130427-supplementary.pdf]

Supporting Information

Enhancing MoS_2 Field-Effect Transistors through Self-Assembled Monolayers: A Strategy to Mitigate Contact Interface Scattering and Boost Device Performance

Li Cao[1], Junqing Wei[1], Shirong Wang, and Guoxuan Qin**

L. Cao, S. R. Wang

Tianjin University, School of Chemical Engineering and Technology, Tianjin, 300072, China, Collaborative Innovation Center of Chemical Science and Engineering, Tianjin, 300072, China

Email: wangshirong@tju.edu.cn

J.Q. Wei, G.X. Qin*

School of Microelectronics, Tianjin Key Laboratory of Imaging and Sensing Microelectronic Technology, Tianjin University, Tianjin 300072, P. R. China

Email: gqin@tju.edu.cn

Li Cao and Junqing Wei contribute equally to this study.

Keywords: Dipole Moment, Field-Effect Transistors, MoS_2, Mobility, Phonon Scattering, Self-Assembled Monolayers

Modification of SiO_2

The SiO_2/Si substrates were sonicated in acetone, isopropyl alcohol, and ethanol for 20 min before being boiled in ethanol. The substrates were treated with an oxygen plasma cleaner at 80W for 10 min after nitrogen drying to eliminate organic contaminants and create a hydroxyl-terminated surface. Herein, SiO_2 functionalized by PFPA, ABPA, and ODPA organo-layers is denoted PFPA-SiO_2, ABPA-SiO_2, and ODPA-SiO_2, respectively.

PFPA-SiO_2: Substrates were immersed in 1 mM PFPA toluene solution at 100 °C for 24h. The samples were then washed with toluene for 5 min under sonication, rinsed with a stream of ethanol, and dried with a nitrogen flow.

ABPA-SiO_2: Substrates were immersed in 1mM APPA aqueous solution at 85 °C for 48 h. The samples were then washed with ultrapure water for 5 min under sonication, rinsed with a stream of ultrapure water, and dried with a nitrogen flow.

ODPA-SiO_2: Substrates were immersed in 1 mM ODPA toluene solution at 100 ℃ for 24h. The samples were then washed with toluene for 5 min under sonication, rinsed with a stream of ethanol, and dried with a nitrogen flow.

Computational methods

We have employed the Vienna Ab Initio Package (VASP) [36,37] to perform all the density functional theory (DFT) calculations within the generalized gradient approximation (GGA) using the PBE [38] formulation. We have chosen the projected augmented wave (PAW) potentials [39,40] to describe the ionic cores and take valence electrons into account using a plane wave basis set with a kinetic energy cutoff of 500 eV. Partial occupancies of the Kohn−Sham orbitals were allowed using the Gaussian smearing method and a width of 0.05 eV. The electronic energy was considered self-consistent when the energy change was smaller than 10^{-7} eV. A geometry optimization was considered convergent when the force change was smaller than 0.001 eV/Å. Grimme's DFT-D3 methodology [41] was used to describe the dispersion interactions. The Brillouin zone integral uses the surfaces structures of Gamma-centered 3×2×1 point sampling, and the supercell (9 Å×10Å ×24Å) is quite large for avoiding image interaction of ABPA molecular. A post-processor, the PHONOPY Code [42], of first-principles for performing phonon dos. The first layer of SiO_2 slab and the ABPA were considered to calculate the phonon properties.

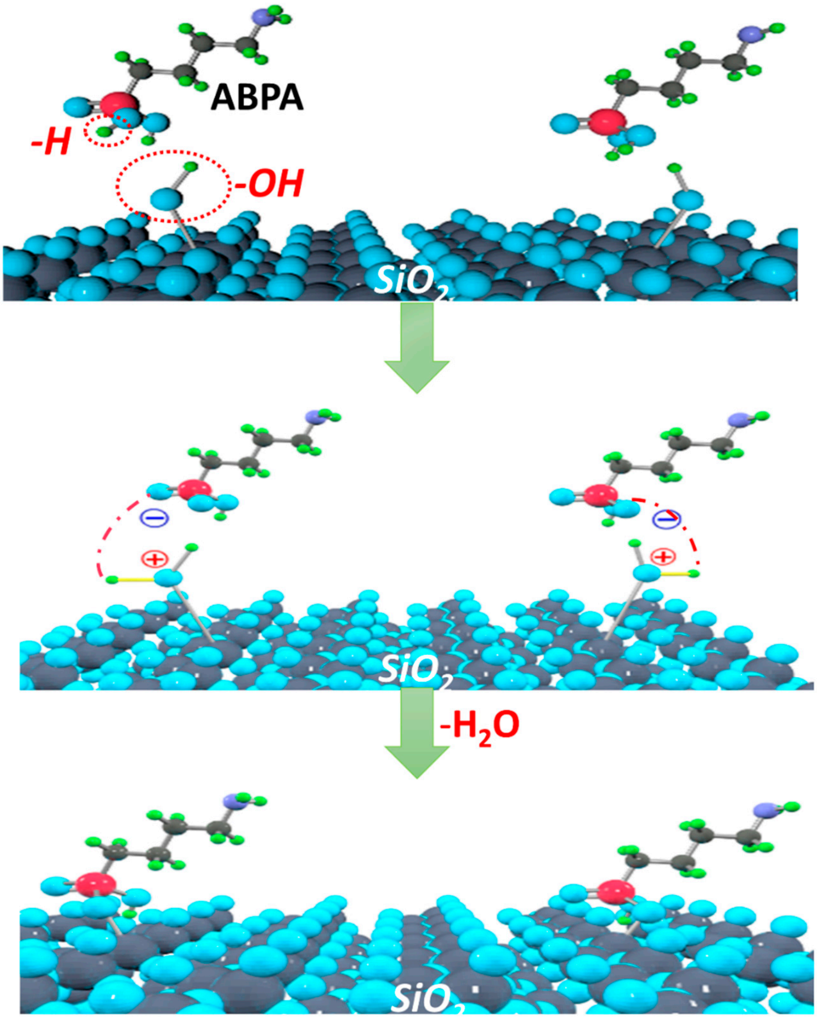

Figure S1. Schematic diagram of the reaction mechanism of phosphonic acids with the hydroxyl groups and bridging oxygen on SiO2 surface.

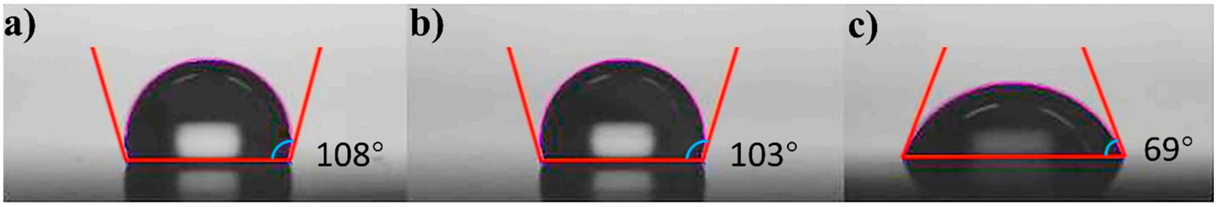

Figure S2. Contact angles of water droplet on (a) ODPA, (b) PFPA, and (c) ABPA-treated Si/SiO_2 substrates.

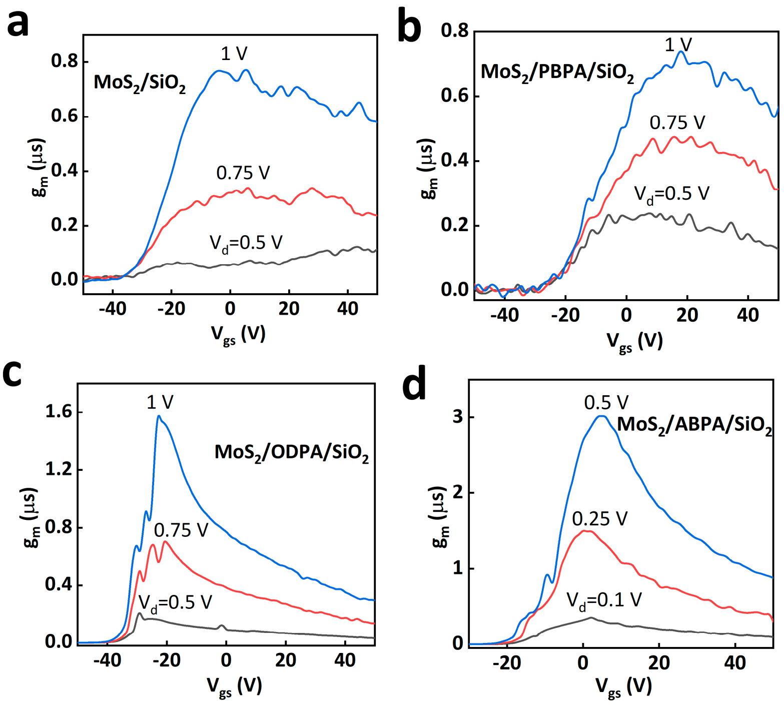

Figure S3. Transconductance values as function of V_g with varying V_d. a PFPA modified device, b ODPA modified device, c ABPA modified device.

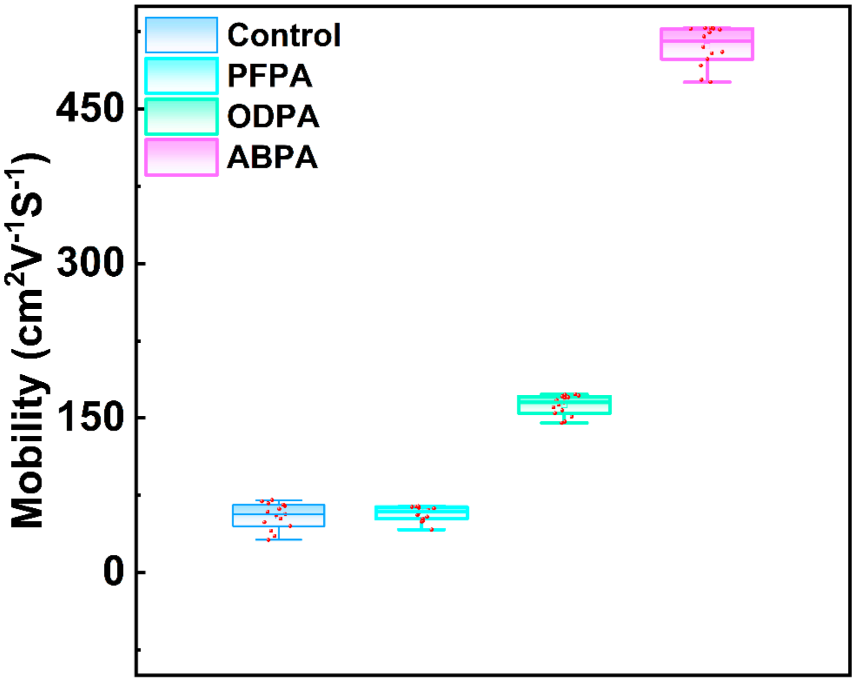

Figure S4. The averaged mobilities with standard deviations of every 15 SiO_2/MoS_2-FETs (without SAMs), SiO_2/PFPA/MoS_2-FETs, SiO_2/ODPA/MoS_2-FETs, SiO_2/ABPA/MoS_2-FETs, respectively.
